# Modeling climate variability and global sugarcane production: Empirical consideration for collective policy action

**DOI:** 10.1016/j.heliyon.2024.e40359

**Published:** 2024-11-16

**Authors:** Hepziba Headley, Stephan Moonsammy, Harold Davis, Devin Warner, Ashley Adams, Temitope D. Timothy Oyedotun

**Affiliations:** a Department of Environmental Studies Faculty of Earth and Environmental Sciences, Guyana; b Department of Geography Faculty of Earth and Environmental Sciences, University of Guyana, P. O. Box 10 1110, Turkeyen Campus, Greater Georgetown, Guyana; c Hydrometeorological Service, Ministry of Agriculture, Georgetown, Guyana

**Keywords:** Climate variability, Sugarcane production, Rainfall, Temperature

## Abstract

The potential impacts of climate change and sugarcane production is well documented in the literature but majority of the studies have focused on models that look at national level impacts. This paper presents a global impact model on sugarcane production due to variations in temperature and rainfall with the intention to observe the collective challenges that sugarcane production is faced with across the world. The study conducted a trend analysis with time series data for sugarcane production, productivity per hectare of sugarcane lands, annual temperature and annual rainfall recorded for the top sugarcane exporters across the world. The study also developed a panel regression model to empirically establish the relationship between production levels and temperature and rainfall. The findings of the study showed that production levels are increasing in some countries while declining in others. Cyclical patterns of production was also observed that seem to vary with cyclical patterns of rainfall. The regression model showed a positive relationship between production and rainfall where a 1 % increase in rainfall can result in a 0.113 % increase in sugarcane production. The model also showed a negative relationship between production and temperature where a 1 % increase in temperature can result in a 0.176 % decrease in sugarcane production. The main conclusion drawn is that as global temperatures continue to increase, then there will be a global decline in the sugarcane industry. This global model for sugarcane and climate change is geared towards showing the collective impacts of climate change experienced by different countries and to encourage from an empirical standpoint, more collective policy actions to protect the industry as a global market.

## Highlights

• Cyclical production trends for sugar cane aligned with similar trends for rainfall.
• Several countries are showing increasing production volumes for sugar cane as a result of climate smart agriculture.
• The models I study shows a 1 % increase in temperature will result in a 0.17 % decrease in global sugar production.
• Collective policy actions are needed to address pertinent climate production issues faced by numerous countries.

## 1 Introduction

Globally, climate change is widely considered as one of the main threats to the earth's ecosystem [1]. Climate change refers to the state of the climate identified by changes in the variability of its properties over a long period of time, while climate variability is the way aspects of the climate, such as temperature and rainfall differ (fluctuate) from their average [2,3]. The profound effect of climate change has increased over the years straddling to all aspects of human society [4]. Ever since the industrial revolution, anthropogenic activities have caused an increase in the Earth's average temperature by 0.9 °C due to greenhouse gas emissions in the atmosphere. Additionally, the temperature is expected to rise to 1.5 °C by 2050 with the current rate of change that is occurring [5,6]. Increases in temperature and precipitation have caused an increase in extreme events such as floods, droughts, irregular patterns of precipitation, heat waves etc. [7,8]. These events all have the potential to negatively impact agriculture production globally [9,10]. Climate variability and change will continue to threaten agriculture production and global food security [11]. The literature shows that increasing temperatures and erratic precipitation will further exacerbate global food security issues as a result of the expected impacts on agricultural production [[12], [13], [14]]. The influence of climate change is expected to not only have an impact on crop production, but also hydrologic balances, input supplies, and other components of agricultural systems [15].

*Saccharum officinarum l.*, commonly known as sugarcane, is a perennial grass that has proven adaptable to a wide range of climatic environments [[16], [17], [18], [19]]. The major sugarcane-producing countries lie in the Asia Pacific, South America, and Caribbean regions. Sugarcane is a climate-sensitive crop requiring the suitability of climatic parameters for high productivity [20]. This therefore means that variation in these climatic parameters can affect commercial sugarcane production on a global scale. Sugarcane contains sugar, water, fiber, and wax which can all be processed into various commercial coproducts [21,22]. On a global scale, sugarcane is a valuable agriculture commodity and is the main agriculture output for the economies of many tropical developing countries [20,23]. It is considered as one of the most important staple foods as it is a significant source of sugar often used in a high volume of processed food products and pharmaceuticals [24]. Industries stemming from the sugarcane industry include sugar, rum, molasses and biofuel. Within the past thirty years, the potential of sugarcane in rum production eventually branched into the renewable energy realm as the ethanol and bagasse from sugarcane is now used as sources of biofuel.

According to the literature, temperature and rainfall are critical determinants of sugarcane production [[25], [26], [27]]. Various agricultural regions worldwide are affected by reduced water availability due to rising temperatures. This ultimately has a negative influence on crop yield [20, [[27], [28], [29]], 30]. Evidence from research shows that the most notable abiotic stresses affecting sugarcane development and productivity for all major sugarcane producing countries is water deficit [22,[31], [32], [33]]. Additionally, many sugarcane producing countries experiences the problem of heat stress resulting from temperature increases [32,34,35]. The optimal temperature for the different phases of sugarcane varies and its effect on the plant's growth and development is essential to production. Temperature stress results in reduced sugarcane growth, quality, productivity, physiology, biochemistry, and longer growing seasons especially in the subtropics [32,[36], [37], [38]]. High temperatures result in drought, a primary abiotic stress causing poor growth, especially for the tillering growth phase. Studies done in South Florida USA revealed that sugarcane is negatively impacted by high temperatures which has caused a lengthening of the growth period for better quality [4,16]. Cardozo and Sentelhas [39] conducted a study in Brazil where they concluded that limited knowledge and understanding exists around the characteristics of sugarcane growth and temperature and the cultivars, which specifically has an adverse impact on the process of the ripening stage of sugarcane.

With respect to rainfall variations, assessing the way in which the irregularity in rainfall distribution affects sugarcane production is of great importance to managing the consequences of it. Sugarcane has a high-water requirement that varies with each development stage. The negative impacts of waterlogging are greatest in the early development phase. The irregular rainfall pattern that leads to waterlogging in sugarcane, reduces the yield by 15–45 % [40]. Moreover, the stage of development, the conditions of the environment, water stress duration and genotypes are responsible for the extent of damage from waterlogging [41]. Similarly, sugarcane growth stages need different amounts of rainfall for development and plant growth to occur. Additionally, the overall response to waterlogging is captured by the negative impact of reduced productivity of 80 % [42]. The crop water requirement for sugarcane is between 1100 and 2000 mm [43,44]. About two-thirds of the water used for sugarcane production is rainwater and the sugar industry is leading the way for optimum recycling [45]. The relatively drought-resistant sugarcane crop reduces production because of water stress and can withstand short flood periods [46]. A recent study in Upper Gangetic Plain (UGP) in India highlighted such importance by assessing the weekly, monthly, seasonal and annual trends of rainfall variability which revealed that overall, there was a decline in the amount of rainfall necessary for sugarcane growth and productivity [47]. The tillering and grand growth phases are directly impacted by drought and proved to be a fundamental limitation, therefore, strategies for mitigating the adverse effect are key to improving productivity [48,49] (Guntukula, 2019; Scarpare et al., 2016).

The demand for sugarcane and its coproducts (sugar and ethanol) therefore means that understanding and managing environmental risks to sugarcane production is of utmost importance. Because of the importance of sugarcane as an agribusiness commodity, it is imperative that the sugarcane producing nations and businesses throughout the world gain a good understanding of the relationships between climate variations and sugarcane production. This paper therefore seeks to empirically analyse the relationship between variations in precipitation and temperature and the global production of sugarcane. Time series graphs will be used to outline any trends in global sugarcane production as it relates to temperature and precipitation variations for each selected country. A panel regression model will be done to determine the degree in which the dependent variables, being the global outputs of sugarcane will be affected by rainfall and temperature.

## 2 Theoretical Framework

The main objective of this study is to establish an empirical relationship between sugarcane productions globally and changes in temperature and rainfall experienced in the main sugar producing countries. The study adopted a simple linear model (Eqn. (1)) with the functional form:

(1) $$\text{Yti}=f\left(X_{1},X_{2}\ldots \;X_{a}\right)$$

Where, Yti is the global production of sugarcane for a given time period (t) and across (i) number of countries. From this basic functional form, the model can then be specified as:

(2) $$\text{Yti}=\beta_{0}+\beta \text{aXt},i+e$$

Where β_0_ is the constant coefficient, βa represents the vector of exogenous coefficients specified for the model, X_t,i_ represent the vector of exogenous variables observed over the time (t) and for the individual countries (i) and (e) represents the error term. The econometric model specified will therefore look at the effects of the exogenous variables for annual rainfall variation and temperature on the production output of sugarcane (Eqn. (2)) for the top sugarcane producing countries in the world so that:

(3) $$\mathrm{Ln}\;\text{Yti}=\beta_{0}+\beta_{1}{\text{LnX}}_{1}\left(t,i\right)+\beta_{2}{\text{LnX}}_{2}\left(t,i,\right)+e$$

Where, X_1_(t,i) is the exogenous variable for rainfall across a time (t) for the individual country (i) and X_2_(t,i) is the exogenous variable for temperature across a time (t) for the individual country (i). A monotonic transformation was conducted (Eqn. (3)) by taking the natural logarithm on both sides of the equation to transform the functional form into a log-log linear model. This sort of transformation allows for the coefficient values estimated to be interpreted as elasticities. The model coefficients can therefore be interpreted as a percentage change in the dependent variable as a result of a percentage change in the independent variables.

## 3 Methodology

The purpose of this paper is to explore the trends in the production of sugarcane in terms of volume and productivity per hectare against the variations in temperature and rainfall for the countries that are considered the top producers of sugarcane globally. Quantitative secondary data on production, yield per hectare and area harvested for sugarcane globally were collected for eleven (11) countries with significant sugarcane export markets globally. Time series data were collected for the top ten sugarcane producing countries in the world which are Brazil, India, China, Thailand, Pakistan, Mexico, Colombia, Indonesia, Philippines, and United States of America along with the largest producer from the Caribbean region which is Guyana. The data were sourced from FAO Stat and the World Bank data websites. The secondary data was collated, cleaned and logarithmic variables were computed and then organized into a stacked panel dataset on Microsoft Excel and analyzed on the Gnu Regression, Econometrics and Time-Series Library (GRETL) software.

The time series data were presented as line graphs to observe time series trends and a Weighted Least Squares (WLS) panel regression model was developed. Time series graphs for rainfall, temperature, production and yield for each country was created to illustrate and compare the trends between these variables across the countries outlined for this study. The panel regression model was performed to establish an empirical relationship of how temperature and rainfall changes affects sugarcane production globally. The Weighted Least Squares model using logarithmic variables was implemented to adjust for any heteroscedasticity, autocorrelation or collinearity issues with the data. Tests for cross sectional dependence was also conducted to assess for potential spatial effects on the model.

## 4 Results and discussion

The time series trends for production and yield per hectare of sugarcane for the selected countries were presented on a time series chart alongside temperature changes and rainfall for the respective countries (see Fig. 1, Fig. 2, Fig. 3, Fig. 4, Fig. 5, Fig. 6, Fig. 7, Fig. 8, Fig. 9, Fig. 10, Fig. 11). From the figures presented, several key trends were observed.

**Fig. 1:**
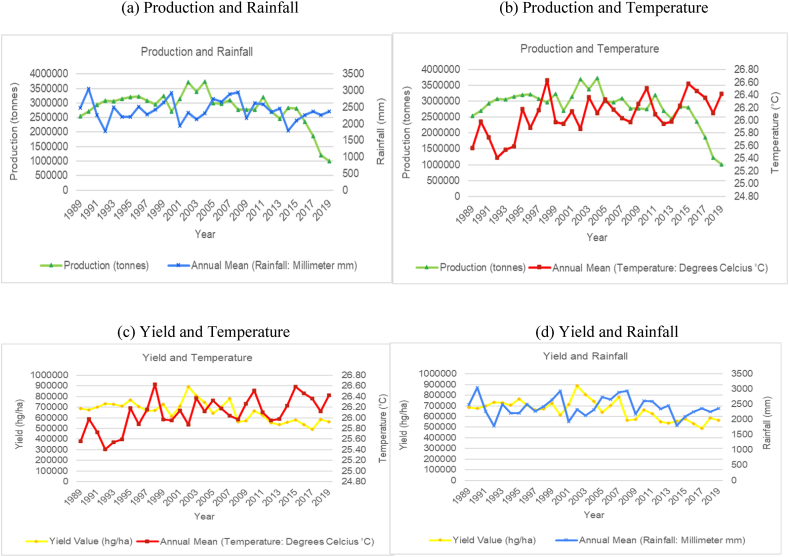
Sugarcane production and yield against temperature and rainfall for Guyana.

**Fig. 2:**
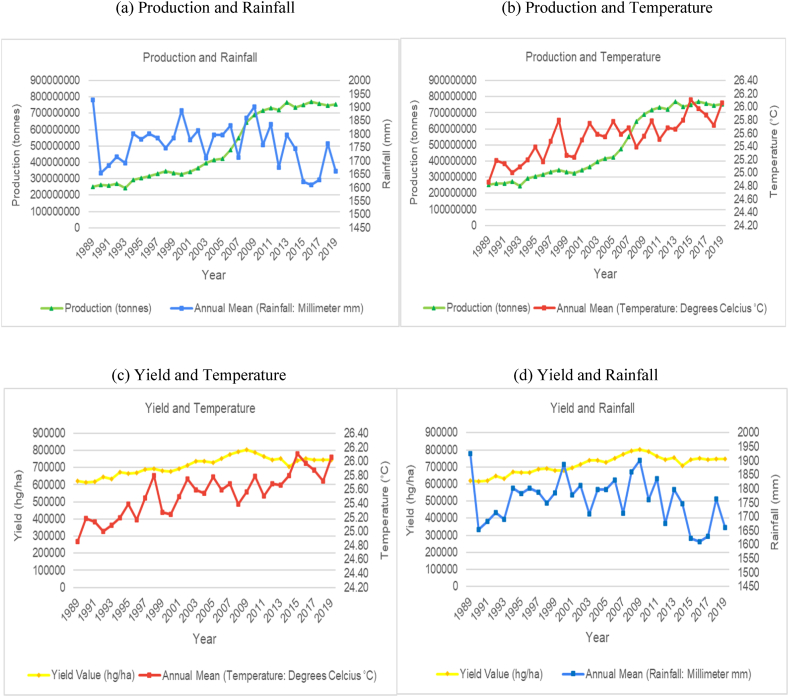
Sugarcane production and yield against temperature and rainfall for Brazil.

**Fig. 3:**
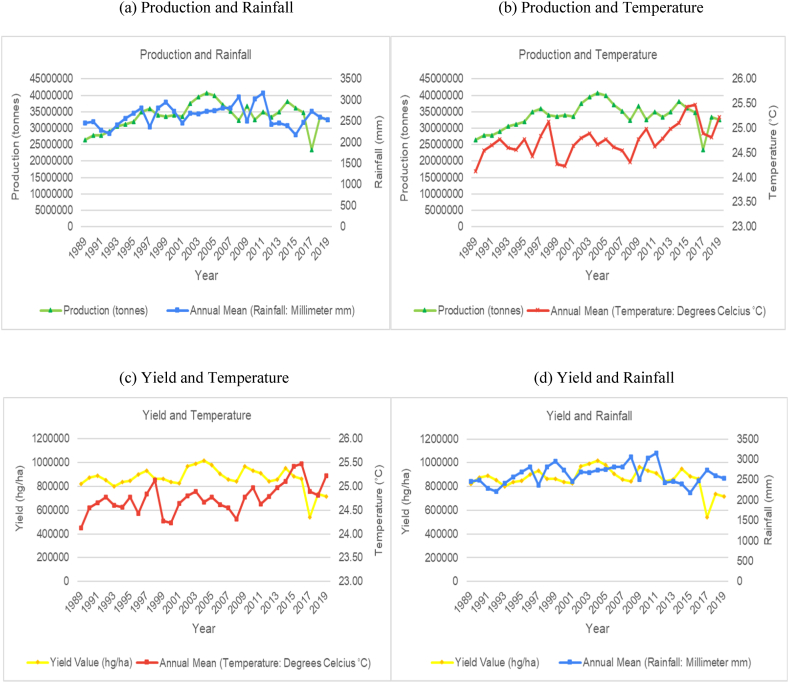
Sugarcane production and yield against temperature and rainfall for Colombia.

**Fig. 4:**
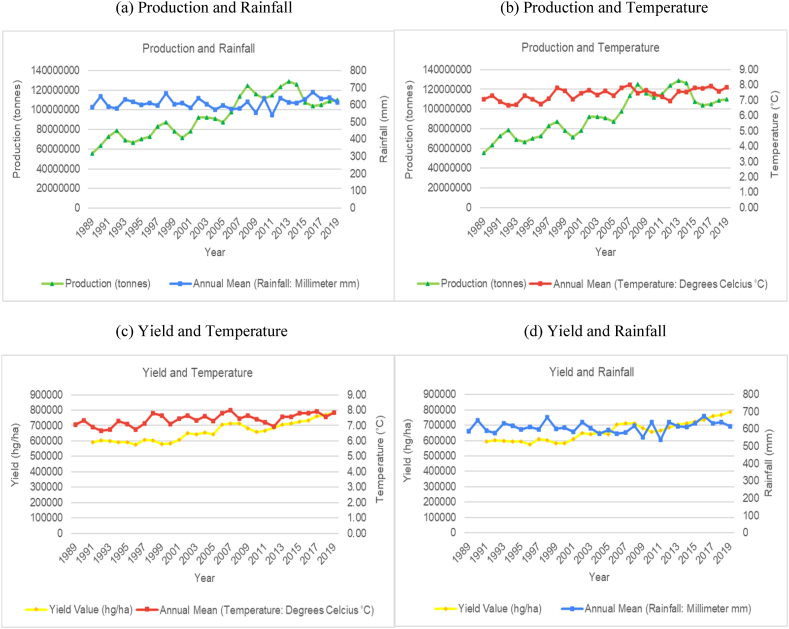
Sugarcane production and yield against temperature and rainfall for China.

**Fig. 5:**
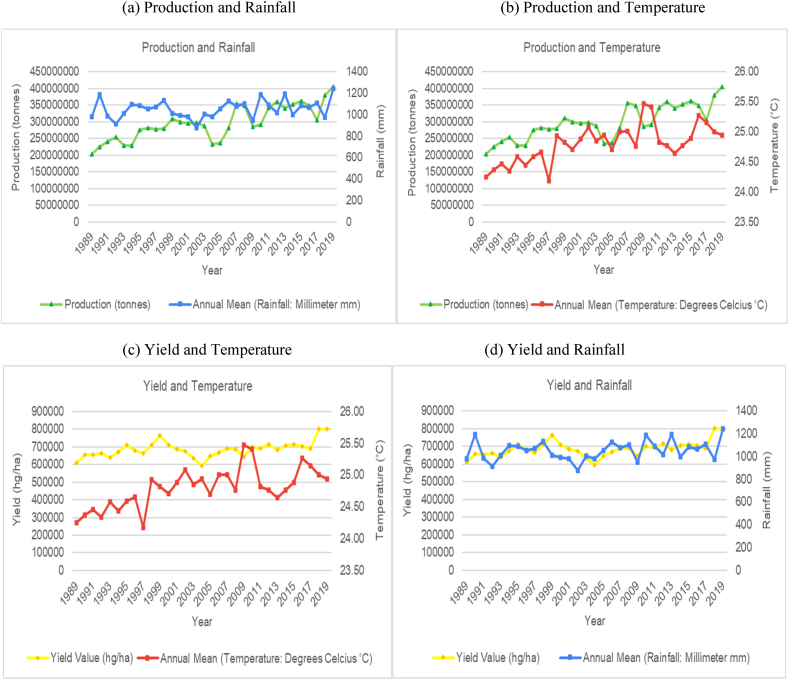
Sugarcane production and yield against temperature and rainfall for India.

**Fig. 6:**
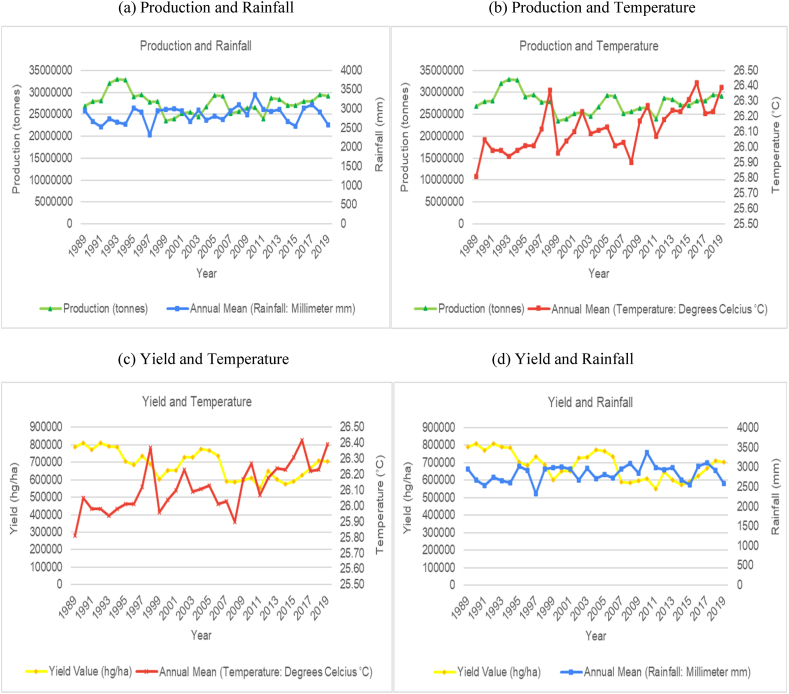
Sugarcane production and yield against temperature and rainfall for Indonesia.

**Fig. 7:**
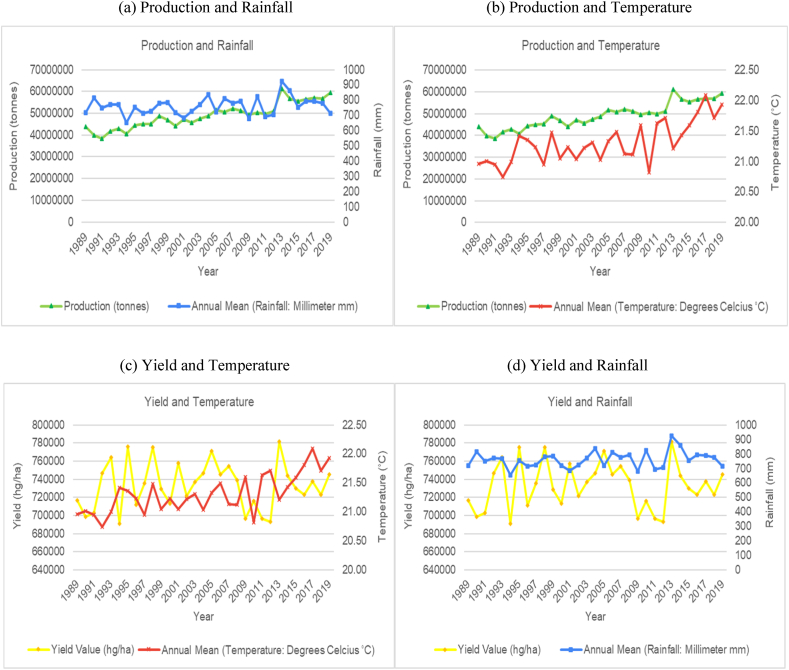
Sugarcane production and yield against temperature and rainfall for Mexico.

**Fig. 8:**
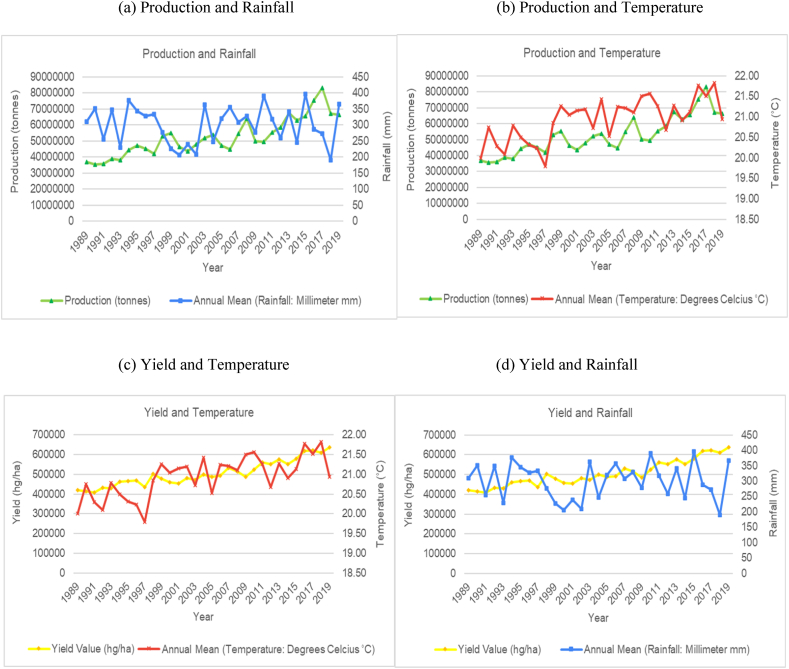
Sugarcane production and yield against temperature and rainfall for Pakistan.

**Fig. 9:**
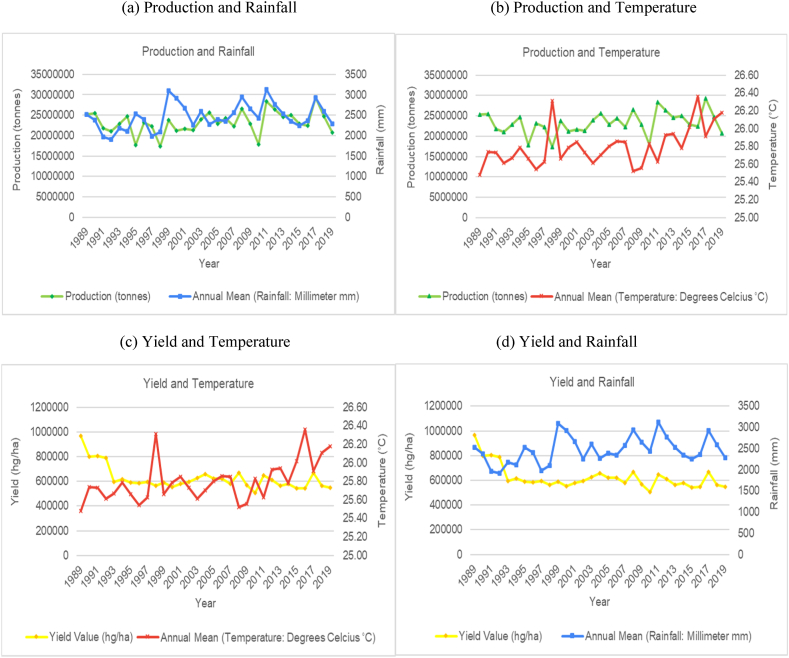
Sugarcane production and yield against temperature and rainfall for Philippines.

**Fig. 10:**
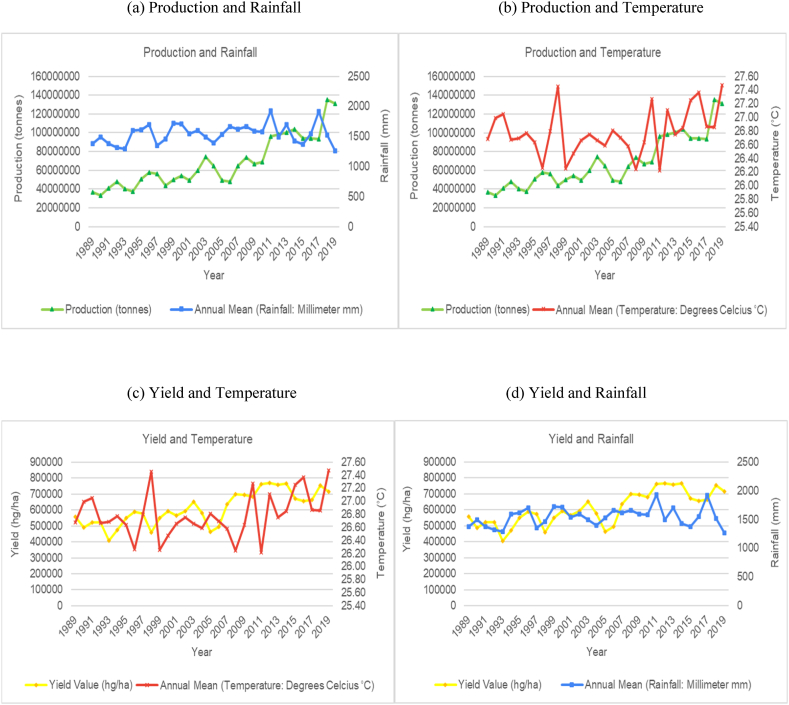
Sugarcane production and yield against temperature and rainfall for Thailand.

**Fig. 11:**
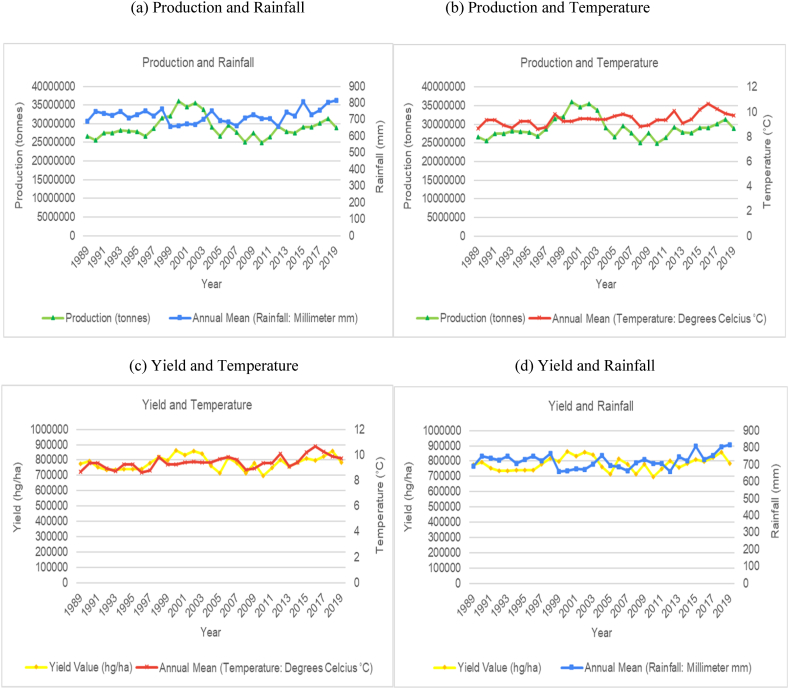
Sugarcane production and yield against temperature and rainfall for the USA.

For the smallest producing country outlined in the study, Guyana showed an increasing and cyclical trend for its annual temperature and a horizontal and cyclical trend for its rainfall (Fig. 1). In terms of its sugar production, the country saw a marginal increase in sugar production and yield per hectare between 1989 and 1999, then a sharp increase from 2000 to 2005. Since 2005, sugar production has been steadily declining. Overall, the data for Guyana shows a decrease of 71.4 % in the production output of sugar for the country. Though the overall trend does indicate a change in production that can be attributed to varying climatic factors, the sharp decline in its production in recent years is mostly attributed to the country's closure of several of its sugar estates because of the unsustainable labour costs in the country resulting in sugar industries in the country operating at a loss.

With respect to the largest producer, Brazil has shown an overall increasing trend in average annual temperatures with annual rainfall having a slight increasing cyclical trend (Fig. 2). Though there is a slight increasing trend for temperature, the data shows the average annual temperatures having peaks and troughs across its time series. However, the overall trend for sugarcane production and yield per hectare is an increasing trend. It is worth noting that Brazil average annual rainfall over the time period ranged from 1600 mm–1950 mm which is double that of some of the other countries assessed for this study. In spite of the variability in temperature and rainfall, Brazil has managed to more than triple its production and yield per hectare of sugarcane over the time series. Brazil's sugar production does not seem to be affected by variability in temperature and rainfall presently. The boost in Brazil's productivity can be attributed to its climate smart agronomy practices and policies and the rapid expansion in the country's biofuel industry [50,51].

Similar to Brazil, Colombia had an increasing trend with its average annual temperature and a cyclical trend with its average annual rainfall as the two countries share similar climatic and ecological spaces (Fig. 3). Unlike Brazil, sugarcane production and yield per hectare for Columbia shows periods across the time series with an upward trend and periods with a downward trend. Colombia's production and yield per hectare had a sporadic downward trend from 2011 onwards. Comparing the trends between rainfall patterns and the sugarcane production variables showed that the volume of rainfall in Columbia is directly impacting production levels. Colombia's rainfall volumes are higher than Brazil ranging from 2400 mm–3000 mm annually. The data trends show that the years where rainfall levels are closer to the 3000 mm upper bound, production levels would show a trough and when rainfall levels are closer to the 2400 mm lower bound, production variables would show a peak. For example, in 2007 and 2010, average annual rainfall reached 3000 mm while sugarcane production dropped from 35 million tonnes the year prior to 31 million tonnes for the both years. Whereas, average annual rainfall in 2009 dropped to 2600 mm and sugarcane production for this year reached up to 36 million tonnes.

China is another major global producer of sugarcane. Interestingly, there is little variation in average annual temperatures and annual rainfall totals (Fig. 4). Temperature variations throughout the time series (1989–2019) have only fluctuated within the 7–8 °C range, while annual rainfall totals were constantly between 550 and 700 mm. The overall trend for sugarcane production and yield per hectare in China has shown an increasing trend, with peak production from 2009 to 2015, after which, a decline was observed. From the graphs, there seems to be no correlation between rainfall and temperature on production levels. As the second largest producer of sugarcane, India has shown an increasing trend in average annual temperature and a cyclical trend for average annual rainfall (Fig. 5). The graphs illustrate that sugarcane production and yield per hectare for India shows periods across the time series with an upward trend and periods with a downward trend. For the most part, comparison of the trends in temperature and rainfall patterns with sugarcane production showed that the temperature and volume of rainfall in India is directly impacting production levels. The data trends show that the years where rainfall levels are closer to the 1200 mm upper bound, production levels are generally lower. Similarly, when temperature levels are closer to the lower bounds (around 24.5 °C), production levels peak, and when temperature levels are closer to the higher bounds (25.5 °C), production levels show a trough. The lower bound of temperature is also associated with higher productivity.

In South East Asia's Oceania region, Indonesia showed an increasing trend with its average annual temperature and a cyclical trend with its average annual rainfall (Fig. 6). The graphs show that sugarcane production and yield per hectare for Indonesia has a downward trajectory. The late 1980's and early 1990's showed periods of peak production and productivity, after which, a moderate downward trend was observed. Similar to India and Columbia, the data trends show that the years where rainfall levels are closer to the upper bound (3500 mm in this case), production showed a trough, and when rainfall levels are closer to the lower bound (2500 mm), production levels peaked. For Indonesia, when rainfall fall within a range of 2800 mm–3000 mm, production stays within a range between 23 million tonnes to 26 million tonnes as observed for the years 1999–2009. However when rainfall volumes are under 2800 mm, there is an increase in production between 27 million and 33 million as observed between 1989 and 1998, in 2013 and 2019. Though it is a rare event according to the data, if rainfall in Indonesia goes above 3300 mm, then sugarcane production slightly decreases, as observed in 2009 when rainfall reached a high of 3500 mm and sugarcane production for that year dropped from 25 million to 24 million tonnes. A similar observation was made for temperature levels. The data trends between temperature levels and production and productivity levels peak were generally sporadic with sharp increases and decreases in temperature from one year to the next. Though these sharp changes in annual temperature do not show a remarkable change in sugarcane production, the patterns of the graph do show an inverse relationship between temperature and production with periods with lower annual temperatures showing higher production volumes and some periods with higher temperatures showing marginal declines in production. The data presented are showing subtle changes from year to year and therefore we cannot definitively conclude on the effect of temperature on production in Indonesia.

On the other side of the globe, in North America, Mexico also showed an increasing trend with its average annual temperature and a cyclical trend with its average annual rainfall (Fig. 7). Unlike Indonesia however, the data trends for Mexico shows a slow but constant increase in production levels (with no major declines) throughout the time series. The graphs show a 50 % increase in sugarcane production for Mexico from the early 1990's to 2019. Comparing yield and temperature trends from graph 7(c) showed a similarity in trend movements between temperature and sugarcane productivity indicating a distinct relationship. The data trends show that when temperature levels were closer to the lower bounds (around 20.7 °C), productivity levels peak, and when temperature levels were closer to the higher bounds (22.2 °C), productivity levels show a trough.

As observed in the data trends for majority of the countries within this study, Pakistan shows a similar trend with respect to annual average temperature and annual rainfall totals (Fig. 8). An increasing trend with its average annual temperature and a cyclical trend with its average annual rainfall can be observed. Unlike the other countries previously discussed, Pakistan's average annual temperature has increased by almost 2 °C over the time series, which represents a much sharper increase in mean annual temperature. Despite the sharp increase in temperature and highly variable rainfall patterns, an overall increase in production and productivity can still be observed over the time series. It is worth noting that when rainfall levels are below 1200 mm, sugarcane production are usually well irrigated. As Pakistan has a low annual rainfall level, the irrigation structure in the country can be attributed to the increasing trend of productivity.

For the Philippines (Fig. 9), a slight increase in annual mean temperature and cyclical rainfall patterns can be observed from the data trends. Interestingly, production levels also seem to be following the cyclical patterns of rainfall. There are periods of rapid decline and increase of sugarcane production over the time series. There is no clear trend as it relates to an overall increase or decline of production levels. However, sugarcane productivity has shown to be on a constant decline.

Similar to the Philippines, Thailand has also shown a slight overall increase in annual mean temperature and cyclical rainfall patterns can be observed from the data trends. The difference however, is that large temperature and rainfall fluctuations has been seen over the time series. Irrespective of these highly variable conditions, production and productivity levels has increased significantly. It is worth noting that Thailand has increased its sugarcane production from forty million tons in 1989 to one hundred and thirty million tons in 2019. This represents an increase of more than 200 % over the time series. It can be seen in the graphs that the higher bounds of the temperature levels (27.5 °C) is associated with lower productivity and vice versa. The same trend is also evident for rainfall on production levels.

Similar to China, the USA has shown little variation in average annual temperatures and annual rainfall totals (Fig. 11). Temperature variations throughout the time series have only fluctuated within the 9–10 °C range, while annual rainfall totals were constantly between 680 and 820 mm. There is no distinct positive or negative trend as it relates to sugarcane production or productivity for the USA. There are periods increasing and decreasing production and productivity levels over the time series, with peak production and productivity occurring from 1999 to 2003, after which, a decline was observed. From the graphs, there seems to be an inverse relationship between rainfall and production parameters. As rainfall levels increase, production and productivity of sugarcane drops and vice versa for the USA. There is no distinct similarities in the trend between temperature and production.

The data trends in the time series observed in the eleven (11) countries used in this study, showed that majority (55 % of the sample) of the countries had an increasing trend in sugarcane production, with some of the countries doubling (India, Pakistan, China) and even tripling (Brazil, Thailand) production levels over the thirty (30) year time series. These countries were able to utilize the climatic parameters through climate smart techniques to enhance production and productivity [20]. A vast majority of the countries showed increasing trends for average annual temperature and cyclical trends for average annual rainfall with relatively consistent annual rainfall totals. For two (2) countries (Philippines and the USA), no distinct trajectory was observed as it relates to an overall increase or decrease in production levels over the time period. Countries like Guyana and Columbia showed a decline in production levels during the latter portion of the time series. This is attributable to various factors. For Guyana, the sharp decline in production and productivity was due to the closure of several sugar estates. This can be viewed as a socio-economic issue that led to the decline. In the case of Colombia however, the decline in production levels was mainly due to the temperature and rainfall variations. The data trends show that when rainfall totals are at its upper bound (3000 mm) production drops significantly, and peaks when rainfall totals are at the lower bound (2400 mm). This was also observed for temperature fluctuations. The decline in production levels in Colombia started after a sharp steady increase in annual mean temperature within the last decade. The trends observed for the major sugarcane producing countries and Guyana follows the pattern outlined in recent research which shows that climate variability has both positive and negative impacts on sugarcane production [5,6].

The study did not take production at the community level across countries into consideration, which may have shown different results as it relates to localized impacts caused by climate change. The increasing trends in sugarcane production and the productivity per hectare observed in the time series can be attributed to several factors such as improved agronomic practices and incorporation of climate smart technology. The overall trends does tell a story of variation existing in sugarcane production that can be attributed to variation in climatic factors. As a means of further expounding on this, the panel regression modeling was done to give some empirical merit to the trend analysis observations.

### 4.1 Panel regression model

The weighted least squares panel regression model was used to establish an empirical relationship between temperature and rainfall changes and the production volumes of sugarcane. A log-log model was implemented therefore the coefficients could have been interpreted as elasticities. The panel regression model results are presented in Table 1 and shows that a 1 % increase in rainfall will increase productivity by 13.5 % and 1 % increase in temperature will decrease productivity by 17.6 %. All the variables in the regression model were statistically significant at 1 %. Globally, the model shows that continued temperature increases will eventually decrease sugarcane production and rainfall decreases impacts sugarcane production positively which is a direct effect of climate change and variability. Sugarcane production is increasing at current levels of temperature and rainfall with mixed variabilities across countries. This corroborates with the literature, for instance, Marin et al. [52] outlined that Brazilian sugarcane producers were concerned about productivity as a result of inconsistent rainfall and temperature. Despite this, Brazil has an increase in productivity primarily due to adaptive agronomic practices and technology implemented in the country. Additionally, a study done by de Medeiros Silva et al. [53] who also used a panel regression model to empirically show the relationship between sugarcane production and climate variability in Brazil showed a similar result where temperature changes negatively impact sugarcane production and rainfall changes positively impacts production.

**Table 1:** Weighted least squares panel regression model on Global sugarcane production.

Variable	Coefficient	Std. Error
Constant	3.44583∗∗∗	0.109320
Ln Rainfall	0.135119∗∗∗	0.0141433
Ln Temperature	−0.175539∗∗∗	0.0217710
Ln Area	1.02469∗∗∗	0.00475821
F-value	17193.14∗∗∗	

Note: ∗∗∗, ∗∗,∗ indicates statistical significance at 1 %, 5 % and 10 % respectively. The model has an F-value significant at 1 %. The R-Squared and Adjusted R-Squared were both 0.993 respectively. The Pesaran CD test has a p-value of 0.535.

The model implemented by de Medeiros Silva et al. [53] was a pooled panel model as this was outlined by the author as the most robust. The model for this study builds upon the robustness of the de Medeiros Silva et al. [53] model by adopting a weighted model that corrects for heteroscedasticity, multicollinearity and autocorrelation and presents a new interpretive viewpoint by having a log functional form. In terms of the model's explanatory power, the R-squared and adjusted R-squared values were over 99% which showed that the model explained majority of the variation in the dependent variable. The F-value was also statistically significant at 1 % which indicates well defined explanatory variables. The Cross-Sectional Dependence (CD) test accounts for the spatial effects, one country climate variability affecting another country of sugarcane production. The Pesaran CD had a p-value of 0.535 which indicates no cross-sectional dependence was present in the model.

Though the findings of this study has been well established theoretically, with small scale models done at the community and national level to empirically validate it. This study presented a full-scale empirical model to further validate the theory. Additionally, there are several policy implications that can stem from the models of this study. The panel regression modeling and trend analysis show distinct patterns in rainfall variation that policy makers can use to implement adaptive strategies. For instance, dependent on the water demand for sugarcane, the reliance of rain fed agriculture and the rainfall levels of the country, the model results can therefore be used to determine the potential investments needed in irrigation systems based on rainfall volume patterns [54]. Countries like Pakistan for instance which shows low rainfall volumes annually and sporadic production volumes can look at more irrigation infrastructure such as drip irrigation in sugarcane production to compensate for rainfall shortages. On the other spectrum, countries like Colombia with high volumes of rainfall can shift investments from irrigation towards more effective drainage and run-off catchment systems.

The main policy implication from the models presented which differs from majority of the climate related studies on sugarcane production is the totality of the impacts measured to the global sugarcane market. The trend analysis shows relative stability in global sugarcane production, the panel model shows that a business as usual approach to climate change will eventually result in a significant decline in sugarcane production and a potential collapse, especially in the smaller sugarcane producing countries. The continued increase in temperatures and the subsequent impacts based on the model of this study therefore shows the need for more collective adaptive policy actions. In order to maintain sugarcane supplies in a globe with an increasing average temperature, new agronomy practices need to be researched in the areas of precision agriculture, permaculture and controlled agro-ecological zones. Most importantly, more collective policy actions are needed such as vertical and horizontal technology transfer, research sharing networks and funding support for developing countries to integrate new adaptive technologies to agronomic systems.

## 5 Conclusion

On a global forefront, agriculture has been affected by climate change significantly in the 20th and 21st centuries. The Intergovernmental Panel on Climate Change (IPCC) last assessment report indicates an increase in average temperature, more frequent heat waves, more stressed water resources, and periods of heavy precipitation will be experienced by most countries. With the unanticipated changes and numerous projected physical and socioeconomic effects of climate change, modelling the impacts to food production is imperative.

This study presented a global model analyzing the empirical relationship between sugarcane production variables and climate variables of temperature and rainfall. Though models presented a strong explanatory power, more empirical work is needed on global models that can include the climate related adaptive strategies for sugarcane production that are implemented in different countries. Where most climate impact modelling on agriculture and food security studies were focused within the specific country of the study, this model does open the research potential of looking at more global production models to demonstrate future food security vulnerabilities and the potential countries where more resources and management interventions may be needed to maintain global supplies. Climate change is a looming threat to all countries and investigating the common impacts with global models can help shape the policy environment needed to implement more collective actions to preserve future food security.

## CRediT authorship contribution statement

**Hepziba Headley:** Writing – original draft, Methodology, Formal analysis. **Stephan Moonsammy:** Writing – original draft, Supervision, Project administration, Methodology, Funding acquisition, Formal analysis, Data curation, Conceptualization. **Harold Davis:** Writing – review & editing, Writing – original draft, Supervision. **Devin Warner:** Writing – review & editing, Writing – original draft. **Ashley Adams:** Writing – review & editing, Writing – original draft. **Temitope D. Timothy Oyedotun:** Writing – review & editing, Supervision, Project administration, Funding acquisition, Conceptualization.

## Data availability statement

The compiled data set will be made available once requested from the authors.

## Declaration of competing interest

The authors declare that they have no known competing financial interests or personal relationships that could have appeared to influence the work reported in this paper.

## References

[1] IPCC (2021, August 9). Climate change widespread, rapid, and intensifying. IPCC; IPCC.

[2] Masson-Delmotte V., Zhai P., Pörtner H.-O., Roberts D., Skea J., Shukla P.R., Pirani A., Moufouma-Okia W., Péan C., Pidcock R., Connors S., Matthews J.B.R., Chen Y., Zhou X., Gomis M.I., Lonnoy E., Maycock T., Tignor M., Waterfield T., IPCC (2018). Global Warming of 1.5°C. An IPCC Special Report on the Impacts of Global Warming of 1.5°C above Pre-industrial Levels and Related Global Greenhouse Gas Emission Pathways, in the Context of Strengthening the Global Response to the Threat of Climate Change, Sustainable Development, and Efforts to Eradicate Poverty.

[3] United Nations Framework Convention on Climate Change (2011, February). Fact sheet: climate change science - the status of climate change science today. https://unfccc.int/files/press/backgrounders/application/pdf/press_factsh_science.pdf.

[4] Zhao D., Li Y.-R. (2015). Climate change and sugarcane production: potential impact and mitigation strategies. International Journal of Agronomy.

[5] Arora N.K. (2019). Impact of climate change on agriculture production and its sustainable solutions. Environmental Sustainability.

[6] Taskinsoy J. (2019). Global cooling through blockchain to avoid catastrophic climate changes by 2050. SSRN Electron. J..

[7] Whitmore J. (2013, October 9). Climate change “unprecedented” by 2050: study. The Conversation.

[8] United Nations (2021). https://www.un.org/en/global-issues/climate-change.

[9] Lobell D.B., Burke M.B. (2010). On the use of statistical models to predict crop yield responses to climate change. Agric. For. Meteorol..

[10] Karimi V., Karami E., Keshavarz M. (2018). Climate change and agriculture: impacts and adaptive responses in Iran. J. Integr. Agric..

[11] Kogo B.K., Kumar L., Koech R. (2021). Climate change and variability in Kenya: a review of impacts on agriculture and food security. Environ. Dev. Sustain..

[12] De Haen H. (2008). Food Security Strategies: Building Resilience Against Natural Disasters Stratégies de sécurité alimentaire: améliorer la résistance aux catastrophes naturelles Strategien für die Sicherung der Ernährung: Stärkung der Widerstandsfähigkeit gegen Naturkatastrophen. EuroChoices.

[13] Wheeler T., Von Braun J. (2013). Climate change impacts on global food security. Science.

[14] Zimmermann B., Kruber S., Nendel C., Munack H., Hildmann C. (2024). Assessing the cooling potential of climate change adaptation measures in rural areas. J. Environ. Manag..

[15] Aydinalp C., Cresser M.S. (2008). *Download Limit Exceeded*. Citeseerx.ist.psu.edu. https://citeseerx.ist.psu.edu/viewdoc/download?doi=10.1.1.475.5379&rep=rep1&type=pdf.

[16] Zhao (2010). Sugarcane response to water-deficit stress during early growth on organic and sand soils. Am. J. Agric. Biol. Sci..

[17] Mirajkar S.J., Devarumath R.M., Nikam A.A., Sushir K.V., Babu H., Suprasanna P. (2019). Advances in Plant Breeding Strategies: Industrial and Food Crops.

[18] Ali S.E., Yuan Q., Wang S., Farag M.A. (2021). More than sweet: a phytochemical and pharmacological review of sugarcane (Saccharum officinarum L.). Food Biosci..

[19] Chen J.C.P., Chung-Chi Chou (1993). https://books.google.gy/books?hl=en&lr=&id=bDIwg2UZ8sYC&oi=fnd&pg=PR19&dq=Chen,+J.+C.+P.,+%26+Chung-Chi+Chou.+(1993).+Cane+sugar+handbook+:+a+manual+for+cane+sugar+manufacturers+and+their+chemists.+John+Wiley+And+Sons.&ots=yrHJWZmqu7&sig=OjMJcSgLiP1xJyLwtp5k5hmj0iI&redir_esc=y#v=onepage&q=Chen%2C%20J.%20C.%20P.%2C%20%26%20Chung-Chi%20Chou.%20(1993).%20Cane%20sugar%20handbook%20%3A%20a%20manual%20for%20cane%20sugar%20manufacturers%20and%20their%20chemists.%20John%20Wiley%20And%20Sons.&f=false.

[20] Kumar A., Choudhary A., Kumar M. (2018). Impact of climate change and their mitigation for better sugarcane production. INTERNATIONAL JOURNAL of AGRICULTURAL SCIENCES.

[21] Srivastava A.K., Rai M.K. (1970). Review: sugarcane production: impact of climate change and its mitigation. Biodiversitas Journal of Biological Diversity.

[22] Srivastava A.K., Rai M.K. (2012). Sugarcane production: impact of climate change and its mitigation. Biodiversitas Journal of Biological Diversity.

[23] de Matos M., Santos F., Eichler P. (2020). Sugarcane Biorefinery, Technology and Perspectives.

[24] O'Hara I.M., Melssen B. (2016).

[25] Sanghera G.S., Malhotra P.K., Singh H., Bhatt R. (2019). Climate change impact in sugarcane agriculture and mitigation strategies. Harnessing Plant Biotechnology and Physiology to Stimulate Agricultural Growth.

[26] Harlianingtyas I., Hartatie D. (2021, March). Modeling of factors affecting the productivity of sugarcane in Jember Regency. IOP Conf. Ser. Earth Environ. Sci..

[27] Shahzad A., Ullah S., Dar A.A., Sardar M.F., Mehmood T., Tufail M.A., Shakoor A., Haris M. (2021). Nexus on climate change: agriculture and possible solution to cope future climate change stresses. Environ. Sci. Pollut. Control Ser..

[28] Kang Y., Khan S., Ma X. (2009). Climate change impacts on crop yield, crop water productivity and food security – a review. Climate change impacts on crop yield, crop water productivity and food security – A review.

[29] Inman-Bamber N.G., Smith D.M. (2005). Water relations in sugarcane and response to water deficits. Field Crops Res..

[30] Liu X., Liu W., Tang Q., Liu B., Wada Y., Yang H. (2022). Global agricultural water scarcity assessment incorporating blue and green water availability under future climate change. Earth's Future.

[31] Ferreira T.H., Tsunada M.S., Bassi D., Araújo P., Mattiello L., Guidelli G.V., Menossi M. (2017). Sugarcane water stress tolerance mechanisms and its implications on developing biotechnology solutions. Front. Plant Sci..

[32] Flack‐Prain S., Shi L., Zhu P., da Rocha H.R., Cabral O., Hu S., Williams M. (2021). The impact of climate change and climate extremes on sugarcane production. GCB Bioenergy.

[33] He S.-S., Zeng Y., Liang Z.-X., Jing Y., Tang S., Zhang B., Yan H., Li S., Xie T., Tan F., Li M. (2021). Economic evaluation of water-saving irrigation practices for sustainable sugarcane production in guangxi province, China. Sugar Tech.

[34] Sonkar G., Singh N., Mall R.K., Singh K.K., Gupta A. (2020). Simulating the impacts of climate change on sugarcane in diverse Agro-climatic zones of northern India using CANEGRO-Sugarcane model. Sugar Tech.

[35] Wahid A., Gelani S., Ashraf M., Foolad M.R. (2007). Heat tolerance in plants: an overview. Environ. Exp. Bot..

[36] Guhan V., Annadurai K., Easwaran S., Marimuthu M., Balu D., Vigneswaran S., Navinkumar C. (2024). Assessing the impact of climate change on water requirement and yield of sugarcane over different agro-climatic zones of Tamil Nadu. Sci. Rep..

[37] Hussain S. (2019). Climate Change and Agriculture.

[38] Hussain S., Khaliq A., Mehmood U., Qadir T., Saqib M., Iqbal M.A., Hussain S. (2018). Sugarcane production under changing climate: effects of environmental vulnerabilities on sugarcane diseases. Insects and Weeds.

[39] Cardozo N.P., Sentelhas P.C. (2013). Climatic effects on sugarcane ripening under the influence of cultivars and crop age. Sci. Agric..

[40] Gomathi R., Gururaja Rao P.N., Chandran K., Selvi A. (2014). Adaptive responses of sugarcane to waterlogging stress: an over view. Sugar Tech.

[41] Muhammad A.A. (2012). Waterlogging stress in plants: a review. Afr. J. Agric. Res..

[42] Misra V., Ansari M.I. (2022). Augmenting Crop Productivity in Stress Environment.

[43] James G.L. (2004). An introduction to sugarcane. Sugarcane.

[44] Hall D.-A. (2015, February 13). https://www.slideshare.net/debbieanhall/sugarcane-cropebook.

[45] International Sugar Organization (2021, May). Press Release (21)22 - ISO's Reply to Natural History Museum Article-a074db.

[46] FAO (2004). http://www.fao.org/ag/AGP/AGPC/doc/GBASE/data/Pf000310.HTM.

[47] Verma R.R., Srivastava T.K., Singh P. (2018). Climate change impacts on rainfall and temperature in sugarcane growing Upper Gangetic Plains of India. Theor. Appl. Climatol..

[48] Guntukula R. (2019). Assessing the impact of climate change on Indian agriculture: evidence from major crop yields. J. Publ. Aff..

[49] Scarpare F.V., Hernandes T.A.D., Ruiz-Corrêa S.T., Kolln O.T., Gava G.J. de C., dos Santos L.N.S., Victoria R.L. (2016). Sugarcane water footprint under different management practices in Brazil: tietê/Jacaré watershed assessment. J. Clean. Prod..

[50] Maroun M.R., La Rovere E.L. (2014). Ethanol and food production by family smallholdings in rural Brazil: economic and socio-environmental analysis of micro distilleries in the State of Rio Grande do Sul. Biomass Bioenergy.

[51] Negra C. (2014). Integrated national policy approaches to climate-smart agriculture. Insights from Brazil, Ethiopia, and New Zealand. CCAFS Report.

[52] Marin F.R., Jones J.W., Singels A., Royce F., Assad E.D., Pellegrino G.Q., Justino F. (2013). Climate change impacts on sugarcane attainable yield in southern Brazil. Climatic Change.

[53] de Medeiros Silva W.K., de Freitas G.P., Coelho Junior L.M., de Almeida Pinto P.A.L., Abrahão R. (2019). Effects of climate change on sugarcane production in the state of Paraíba (Brazil): a panel data approach (1990–2015). Climatic Change.

[54] Parkes B., Higginbottom T.P., Hufkens K., Ceballos F., Kramer B., Foster T. (2019). Weather dataset choice introduces uncertainty to estimates of crop yield responses to climate variability and change. Environ. Res. Lett..

